# Polydopamine-Coated Polycaprolactone Electrospun Nanofiber Membrane Loaded with Thrombin for Wound Hemostasis

**DOI:** 10.3390/polym15143122

**Published:** 2023-07-22

**Authors:** Dapeng Cui, Ming Li, Peng Zhang, Feng Rao, Wei Huang, Chuanlin Wang, Wei Guo, Tianbing Wang

**Affiliations:** 1 Hepatobiliary Surgery Department, The First Affiliated Hospital of Hebei North University, Zhangjiakou 075000, China; 2 Trauma Medicine Center, Peking University People’s Hospital, Beijing 100044, China; 3 Key Laboratory of Trauma and Neural Regeneration, Ministry of Education, Peking University, Beijing 100044, China; 4 National Center for Trauma Medicine, Beijing 100044, China

**Keywords:** trauma, electrospinning, polydopamine modification, thrombin, hemostatic method

## Abstract

Hemorrhagic shock is the primary cause of death in patients with severe trauma, and the development of rapid and efficient hemostatic methods is of great significance in saving the lives of trauma patients. In this study, a polycaprolactone (PCL) nanofiber membrane was prepared by electrospinning. A PCL–PDA loading system was developed by modifying the surface of polydopamine (PDA), using inspiration from mussel adhesion protein, and the efficient and stable loading of thrombin (TB) was realized to ensure the bioactivity of TB. The new thrombin loading system overcomes the disadvantages of harsh storage conditions, poor strength, and ease of falling off, and it can use thrombin to start a rapid coagulation cascade reaction, which has the characteristics of fast hemostasis, good biocompatibility, high safety, and a wide range of hemostasis. The physicochemical properties and biocompatibility of the PCL–PDA–TB membrane were verified by scanning electron microscopy, the cell proliferation test, the cell adhesion test, and the extract cytotoxicity test. Red blood cell adhesion, platelet adhesion, dynamic coagulation time, and animal models all verified the coagulation effect of the PCL–PDA–TB membrane. Therefore, the PCL–PDA–TB membrane has great potential in wound hemostasis applications, and should be widely used in various traumatic hemostatic scenarios.

## 1. Introduction

In the emergency department, trauma-induced hemorrhagic shock is the leading cause of death [1,2,3]. Therefore, rapid and efficient hemostatic first aid measures are essential to save the lives of trauma patients, especially in cases of traumatic bleeding of the liver, spleen, kidneys, and other substantial visceral trauma, as well as bleeding from large vessels such as the aorta, femoral artery, and mesenteric artery [4,5,6,7]. At present, the main hemostatic materials used in the clinic include the gelatin sponge, fibrin glue, polysaccharide hemostatic powder, etc., [8,9,10] whose main hemostatic mechanisms include the following: an adsorbent material, which networks the formation of components in the blood, thereby providing a mechanical structure for the mutual adhesion between platelets in order to promote thrombosis; a compress material, which promotes hemostasis by absorbing water and expanding or promotes mechanical hemostasis by dissolving the adhesion wound; and hemostatic materials, which exert hemostatic effects by delivering procoagulant drugs [11,12,13]. However, these hemostatic materials have certain limitations, such as low hemostatic efficiency, poor biocompatibility, harsh storage, and use conditions, etc., meaning they cannot fully meet the urgent needs of all trauma response first aid [14,15]. Exploring and developing new rapid hemostatic materials with better performance is an urgent clinical need.

Thrombin is a serine protease extracted from human or animal blood. It is a biological factor that plays a major role in the coagulation cascade, activating FXIII and converting fibrinogen in the blood into fibrin to achieve hemostasis [16]. As we all know, thrombin has many advantages: relevance to a variety of bleeding wound types, rapid function, reasonable biocompatibility, and non-toxicity, ease of obtaining and using, etc., which lead to its wide use in capillaries, small blood vessels, and organs to stop bleeding; additionally, it has an excellent hemostatic effect [17,18]. However, on the other hand, because of its near-non-adhesion, when used for bleeding in larger wounds, it is easily washed away by the blood flow, which causes it to lose its hemostatic effect; furthermore, its harsh storage environment (low temperature) further limits its range of applications [19,20]. Therefore, it is important to develop thrombin-loaded membranes or other materials instead of powder-based materials.

Thrombin can be linked by a polymer to convert the thrombin powder into a thrombin membrane for acute hemostasis. Nanofiber membranes prepared using electrospinning technology have the characteristics of large specific surface area, small pore size, and high porosity [21,22]. Because their nanostructure can simulate the structure of the extracellular matrix (ECM), they can be loaded with active ingredients that promote therapy. They also provide air to the wound area and keep the healing environment moist [23]. Chen Kai et al. prepared a novel curcumin-loaded sandwich nanofiber membrane by sequential electrospinning, which can effectively prevent bleeding, inhibit bacteria, and accelerate wound healing [24]. Liu Tao et al. prepared a chitosan/polyethylene oxide/kaolin nanofiber membrane by electrospinning and found that their nanofiber membrane of 10% weight percentage kaolin showed excellent hemostatic performance; it was proven that back wounds on 14-day-old rats were healed without causing any obvious inflammatory reaction [25]. Mirmajidi et al. prepared three layers of chitosan–polycaprolactone/polyvinyl alcohol–melatonin/chitosan–polycaprolactone nanofiber dressings by electrospinning and found that it had good attachment to hydrophilic support cells and effectively promoted wound healing in a full-layer skin resection model of rats [26]. Based on the above advantages, the use of electrospinning technology to transform thrombin powder into a thrombin membrane can produce the structural advantages of an electrospun membrane while maintaining the hemostatic activity of thrombin, producing a synergistic effect for wound hemostasis, which is a new and effective strategy. PCL is often used as a raw material in electrospinning technology. It is a commonly used biodegradable synthetic polymer material that degrades into non-toxic metabolites in vivo [27,28,29]. However, due to the hydrophobic surface structure of pure polymer electrospun membranes, it is difficult for proteins and cells to attach, resulting in low drug loading efficiency, poor cell affinity, and poor biological activity.

In recent years, polydopamine (PDA) coating, as a simple and mild surface modification method, has been shown to improve the adhesion properties of modified biomaterials and has been widely studied by researchers. In particular, 3,4-dihydroxy-L-phenylalanine (DOPA) and lysine-enriched proteins near the patch–substrate interface were found to be the main sources of the wet adhesion properties found in mussels [30,31]. Inspired by the mussel, PDA has a similar molecular structure to DOPA and can be easily deposited on all types of inorganic and organic substrates with controllable film thickness and long-lasting stability [32]. Moreover, the chemical structure of PDA has many functional groups, such as catechols, amines, and imines [33]. These functional groups provide abundant covalent binding sites for desired molecules [34]. Due to its good biocompatibility and its ability to promote cell proliferation [35], PDA has been used in many fields to develop bioactive molecular delivery systems. For example, PDA-coated chitosan membranes have been used to deliver vascular endothelial growth factor (VEGF) in vascular tissue engineering [36]. In addition, PCL–nanocarbon fiber scaffolds coated with PDA have been used to deliver brain-derived neurotrophic factor (BDNF) in nerve repair [37]. However, the study of using PDA surface modification to efficiently load thrombin onto nanofiber membranes for wound hemostasis has not been reported so far.

To this end, in this study, the PCL nanofiber membrane was prepared by electrospinning method, and thrombin was loaded onto the membrane by PDA surface modification. The loading efficiency was evaluated using TB model protein (FITC-BSA). Then, fibroblasts were co-cultured with membranes to evaluate the effects of membranes on fibroblast growth and adhesion. Further, subcutaneous embedding models of SD rats were used to evaluate the biosafety of the membranes in vivo. Then, the in vitro coagulability of membranes was tested by dynamic coagulation test and adhesion test of red blood cells and platelets. Finally, the in vivo coagulability of membranes was evaluated using SD rat liver hemorrhage model and femoral artery hemorrhage model.

## 2. Materials and Methods

### 2.1. Preparation of PCL Fibrous Membranes

A total of 1 g polycaprolactone (PCL, Jinan Daigang Co., Ltd., Jinan, China) was added into 10 mL chloroform (McLean Reagent Co., Ltd., Beijing, China) and N, N-dimethylformamide (McLean Reagent Co., Ltd., Beijing, China) in a mixed solvent (8:2, *v*/*v*), and stirred with a magnetic stirrer until completely dissolved to obtain spinning solution. The viscosity of the electrospinning solution was determined using a rheometer (HAAKE MARS60) and repeated three times. The prepared electrospinning solution was loaded into a 20 mL syringe, and the electrospinning voltage was set to 25 kV, the solution flow rate to 1.0 mL/h, the receiving distance to 16 cm, the temperature to 25 °C, and the humidity to 50%. The PCL fibrous membranes obtained by electrospinning machine was placed in a fume hood for 12 h to allow the solvent to fully evaporate, and then dried in a vacuum oven for 6 h to completely remove residual solvent, then a preliminary PCL fibrous membrane was obtained.

### 2.2. Coating PDA onto PCL Fibrous Membranes

Dopamine (McLean Reagent Co., Ltd., Beijing, China) was dissolved in 10 mM Tris HCl (pH = 8.5) to prepare 2 mg/mL dopamine solution. The PCL nanofiber membrane was placed in a dopamine solution, soaked in the dark for 12 h, washed with deionized water three times, and freeze-dried to obtain the PCL–PDA fibrous membrane.

### 2.3. Characterization of Membranes

Gold was sprayed on PCL and PCL–PDA fibrous membranes, and the morphology of the fibrous membranes was observed using field emission scanning electron microscopy (SEM, HITACHI SU8010, Tokyo, Japan). The surface chemical elements of PCL and PCL–PDA fibrous membranes were analyzed by energy-dispersive spectroscopy (EDS) and elemental mapping. Image-Pro Plus 6.0 software (Media Cybernetics, Rockville, MD, USA) was used to randomly select 200 nanofibers from PCL and PCL–PDA samples to analyze the diameter distribution of nanofibers. The water contact angles of the PCL and PCL–PDA fibrous membranes were measured with a water contact angle tester (OCA20, DataPhysics, Filderstadt, Germany). The elastic modulus and elongation at break of the fabricated scaffolds were measured with a universal testing machine (Model 5848, Instron, Norwood, MA, USA). Each group of samples was repeated three times. The degradation rate of PCL, PCL–PDA, and PCL–PDA–TB fibrous membranes was studied by immersing the membranes (12 × 12 mm) in phosphate buffered saline (PBS) (pH 7.4) at 37 °C. After 3 months, the membranes were taken out, vacuum-dried, and weighed. The weight loss was calculated as the difference between the original weight and the remaining weight. Subsequently, the degradation rate was calculated as the ratio of lost weight to original weight. Each group was tested independently 3 times. A separate sample was used for each data.

### 2.4. Thrombin Loading

A TB solution was prepared with a concentration of 250 U/mL, the PCL and PCL–PDA fibrous membranes were soaked in the TB solution for 12 h, washed with deionized water three times, and freeze-dried to obtain a PCL-TB and PCL–PDA–TB fibrous membranes. The TB loaded on the fiber scaffold was observed by SEM. The surface chemical elements of PCL–PDA–TB fiber membranes were analyzed by energy dispersive spectrometry (EDS) and element diagram.

In order to evaluate the distribution of TB on the membranes, fluorescein isothiocyanate-labeled bovine serum albumin (FITC-BSA) (Solarbio, Beijing, China) was applied as a model drug for TB according to the procedure described above. After FITC-BSA loading, the membranes were harvested, washed with deionized water, and observed using a confocal laser scanning microscope (CLSM) (TCS-SP8, Leica, Wetzlar, Germany). Image-Pro Plus 6.0 software was used to compare the fluorescence intensity of FITC-BSA on the membranes. Six independent fields of view were selected for each group of samples.

### 2.5. In Vitro Biosafety Evaluation

Prepared PCL and PCL–PDA samples with concentrations of 5000, 2500, 1250, 625, 312.5, and 156.25 μg/mL and placed in culture medium for 24 h to make an extract. Cultivated mouse fibroblasts cells (L-929, NCTC) and adjusted density to 1 × 10^5^/well, were inoculated on a 24-well plate. A total of 1 mL of extraction solution was placed on the pore plate. After 24 h, 100 mL was added to the CCk-8 reagent. It was incubated in a cell culture chamber at 37 ℃ for 4 h, and the absorbance of the supernatant was measured at 450 nm on an enzyme-linked immunosorbent assay.

Fibrous membranes were sterilized (UV sterilization) for 1 h, and PBS solution was used to wash three times to remove residual ethanol. The sample was placed in a 24-well plate and inoculated fibroblasts at a density of 1 × 10^5^/well on it. On days 1, 3, and 5, cells were stained with Calcein-AM and cell growth was observed by laser confocal microscopy (OLYMPUS FV1000, Tokyo, Japan). On the third day, the culture medium was removed from the well, the sample was washed three times with PBS solution, fixed with 3% glutaraldehyde for 12 h, dehydrated with gradient alcohol, and the sample was observed using SEM after gold spraying treatment.

### 2.6. In Vivo Biosafety Evaluation

To evaluate the biocompatibility and degradability of fiber membranes in vivo, we anesthetized SD rats with pentobarbital sodium and removed hair on the back. After the incision on the back skin, a 20 mg sample was implanted. In the blank group, only skin incision and suture were performed without embedding any material. The positive control group was filter paper. The skin reaction of each group was observed at 0, 3, 7, and 14 days. After 14 days, the rats in each group were euthanized, and the whole skin tissue of each group was stained with hematoxylin-eosin (HE) to observe the skin pathological changes (especially foreign body reaction and inflammatory reaction) and evaluate the degradation performance of the material. In addition, the shape and size of heart, lung, stomach, liver, spleen, and kidney of blank group and PCL–PDA group were observed. Then, the pathological changes in these organs were observed by HE staining to assess whether PCL–PDA caused substantial damage to various organs after degradation and absorption.

### 2.7. Blood Coagulation In Vitro

Fresh rat blood was extracted and mixed with Anticoagulant citrate dextrose solution (ACD, 20 mM citric acid, 110 mM sodium citrate, and 5 mM D-glucose) at a ratio of 9:1 (*v/v*) to make anticoagulant whole blood for later use. The samples of PCL, PCL–PDA, and PCL–PDA–TB were cut into a square (0.5 × 0.5 cm^2^) of the same size and put into the 12-hole plate. The hole plate of the sample was not added as the control group. A total of 50 μL anticoagulant whole blood drops were added to the surface of each group of samples, and then 10 μL 0.2 mol/LCaCl_2_ solution was added to initiate coagulation. Then the samples were placed in a constant temperature oscillating incubator at 37 ℃ for 10 min, 20 min, 30 min, 40 min, 50 min, and 60 min. After that, 3 mL of deionized water was added to each sample, gently shaken, and left for 5 min. Finally, the absorbance of the supernatant was measured at 545 nm by enzymograph, and the dynamic coagulation curves of each group were obtained. The whole blood clotting index (BCI) is calculated as follows:

BCI = [(A_s_ − A_b_)/(A_c_ − A_b_)] × 100% (1)

where A_s_ is the OD value of the experimental group, A_c_ is the OD value of the control group, and A_b_ is the OD value of the blank hole.

Each group of samples was repeated three times.

### 2.8. Red Blood Cell Adhesion

The PCL, PCL–PDA, and PCL–PDA–TB samples were placed in a 24-well orifice plate. A total of 1 mL of anticoagulant whole blood was dropped in each sample. The 24-well plate was incubated in a 37 °C incubator for 5 min to make the sample fully interact with the blood. Deionized water was then slowly added to the surface of each sample to wash away the red blood cells that had not been captured by the material. After fixation with 3% glutaraldehyde for 12 h, dehydration and gold spraying with gradient alcohol were performed. The morphology and number of red blood cells adhering to the surface of the sample were observed by SEM.

### 2.9. Platelet Adhesion

The procedure of this part of the experiment is similar to that of red blood cell adhesion. The difference was that anticoagulant whole blood was centrifuged at 800 rpm for 10 min, and the supernatant was obtained with a pipette to obtain platelet-rich plasma (PRP), which was then added to the surface of the sample and incubated for 2 h. Finally, SEM was used to observe the number and activation degree of platelets adhering to the surface of the sample.

### 2.10. In Vivo Hemostatic Test

All experimental procedures in this study were carried out in accordance with ethical guidelines and approved by the Animal Ethics Committee of Peking University People’s Hospital (approval number: 2021PHE067). This part of the experiment used 60 SD rats purchased from Beijing Weitonglihua Laboratory Animal Technology Co., Ltd. (License No. SCXK (JING) 2021-0011). Each experimental animal was randomly divided into 5 groups with 6 animals in each group: control group (using ordinary gauze), PCL group, PCL–PDA group, PCL–PDA–TB group, and commercial group (using absorbable soluble hemostatic material, purchased from Qingdao Zhonghui Shengxi Biological Engineering Co., Ltd., Qingdao, China).

Femoral artery hemostasis experiment. The SD rats were anesthetized and fixed in a supine position. The skin and muscle were cut layer by layer from the inner thigh to expose the femoral artery. The femoral artery was punctured with the needle of a 1 mL syringe, then materials of each group were placed at the wound and fully fitted to the wound through appropriate pressure. The time of complete stop of bleeding and the amount of bleeding were recorded.

Liver hemostasis experiment. SD rats were anesthetized, fixed in supine position, shaved, disinfected, and a median abdominal incision about 5 cm long was made, and the skin, muscle, and peritoneum were cut layer by layer. The liver was exposed. After the right lobe of the liver was fully exposed, a scalpel was used to make a cutting wound about 0.5 cm long and 0.2 cm deep on it. The materials of each group were placed in the wound, fully covered, and attached to the liver tissue. The dry cotton ball was applied to the wound on the outside of the material. The time of complete stop of blood seepage and the amount of blood loss were recorded.

### 2.11. Statistical Analysis

All data are expressed as mean ± standard deviation (SDs). SPSS 13.0 statistical software and one-way analysis of variance (ANOVA) were used to analyze the data. * *p* < 0.05, ** *p* < 0.01, *** *p* < 0.001 were defined as all statistical tests were significant, ns meant *p* > 0.05, and there was no statistical difference in the results. Each group was tested independently 3 times. A separate sample was used for each dataset.

## 3. Results

### 3.1. Characterization of Membranes

**Figure 1:**
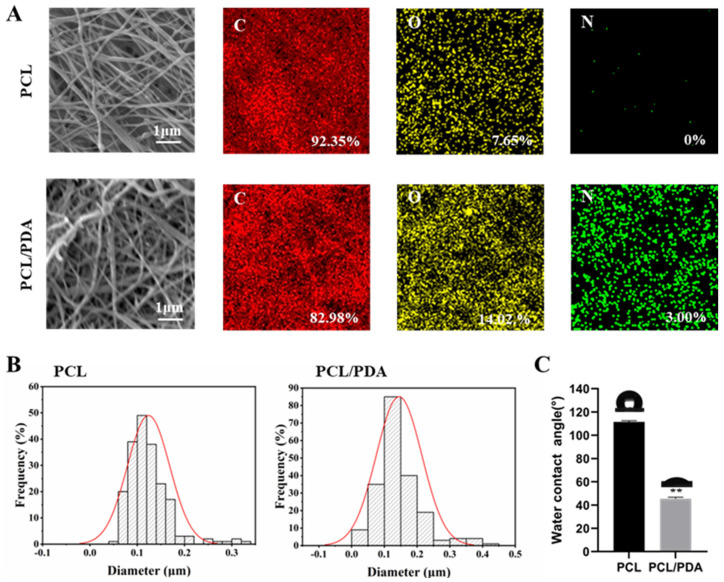
(**A**) SEM and EDS elemental mapping images of PCL and PCL–PDA fibrous membranes. (**B**) Fiber diameter distribution of PCL and PCL–PDA fibrous scaffolds (*n* = 200). (**C**) Water contact angles of PCL and PCL–PDA fibrous membranes (*n* = 3, ** *p* < 0.01).

The viscosity of the initial solution affects the process and result of electrospinning. The viscosity of PCL solution was determined to be 202 ± 2.16 mPa s. Then, the PCL and PCL–PDA nanofiber membranes were observed by SEM. As can be seen from Figure 1A, the distribution of the two scaffolds was uniform and smooth. The surface of PCL fiber membrane was smooth, and the surface of PCL–PDA fiber membrane was rough. The surface elements of PCL and PCL–PDA fiber membranes are also shown in Figure 1A. After PDA coating, the content of N increased from 0 to 3.00%. As can be seen from Figure 1B, the fiber diameter of PCL (0.12 ± 0.04 μm) was close to that of PCL–PDA (0.14 ± 0.07 μm), and there was no statistical difference between them (*p* > 0.05). Figure 1C shows the water contact Angle of PCL and PCL–PDA fiber membranes. After PDA coating, the water contact Angle decreased significantly from 111.47 ± 1.09° to 45.46 ± 1.27° (*p* < 0.01). There was no significant difference in the mechanical properties of the samples before and after the surface coating of PDA and loaded TB (*p* > 0.05) (Figure S1). As shown in Figure S2, there was no significant difference in degradation rate among all groups after 3 months (*p* > 0.05). It can be seen that the surface treatment of PCL nanofibers has no obvious effect on their mechanical properties and degradation.

### 3.2. Loading of Thrombin

**Figure 2:**
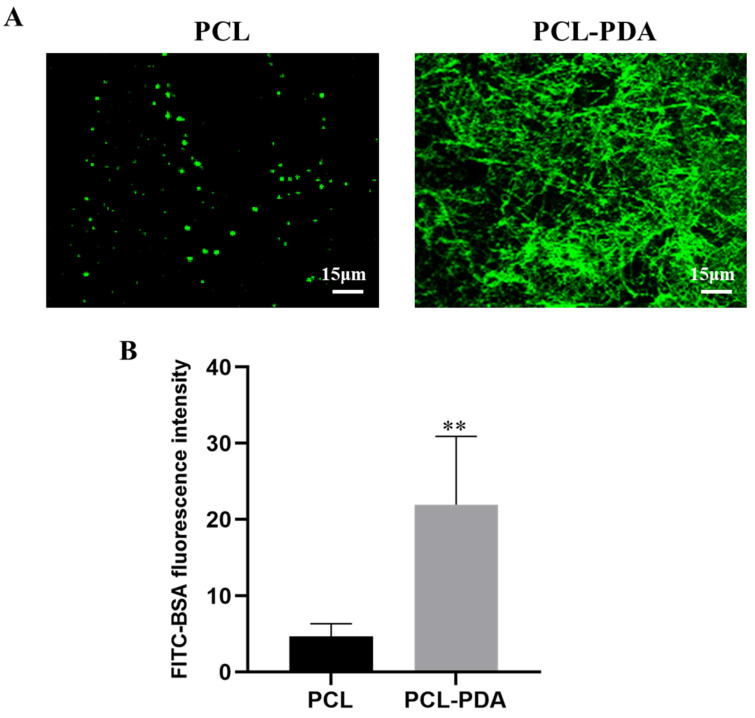
(**A**) CLSM images of FITC-BSA loaded onto PCL and PCL–PDA fibrous membranes. (**B**) Fluorescence intensity of FITC-BAS on PCL and PCL–PDA fibrous membranes (*n* = 6, ** *p* < 0.01).

Figure 2A shows that the fluorescence intensity of the PCL fiber membrane was much lower than that of the PCL–PDA fiber membrane, indicating that the PCL–PDA fiber membrane was loaded with more thrombin model drug (FITC-BAS). By quantification and comparison of fluorescence intensity (Figure 2B), PCL–PDA fiber membrane was much higher than PCL fiber membrane (*p* < 0.01). We also observed the SEM images and surface element distribution of PCL-TB and PCL–PDA–TB fiber membranes loaded with thrombin. As can be seen from Figure S3, the PCL–PDA membrane had more thrombin particles on the fiber surface than the PCL membrane. As shown in Figure S4, the surface of PCL–PDA–TB fiber membrane increased the S element specific to thrombin. The results showed that thrombin was loaded onto the surface of PCL–PDA fiber membrane.

### 3.3. In Vitro Safety Evaluation of Membranes

**Figure 3:**
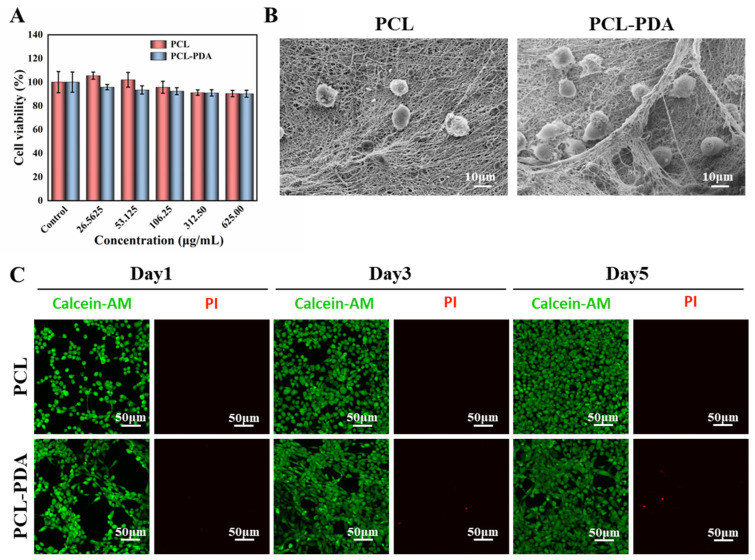
(**A**) Cell viability of PCL and PCL–PDA fibrous membranes incubated with Fibroblasts cells for 1 day. (**B**) SEM images of Fibroblasts cells grown on PCL and PCL–PDA fibrous membranes. (**C**) Representative CLSM images of fibroblasts cells after 1 day, 3 days, and 5 days of culture on PCL and PCL–PDA fibrous membranes.

The detection results of CCK8 are shown in Figure 3A. The survival rate of membranes cultured with different concentrations of PCL and PCL–PDA fiber membranes was more than 90% after 1 day, indicating that both membranes had good biocompatibility. As shown in Figure 3B, in the SEM images after 3 days of culture, more membranes were adhered to the PCL–PDA fiber membrane than the PCL fiber membrane. On days 1, 3, and 5, living cells were colored green by Calcein-AM and dead cells were colored red by PI. There is little red fluorescence in Figure 3C. The results also showed that PCL fiber membrane and PCL–PDA fiber membrane had good cytocompatibility.

### 3.4. In Vivo Biosafety Assessment of Membranes

**Figure 4:**
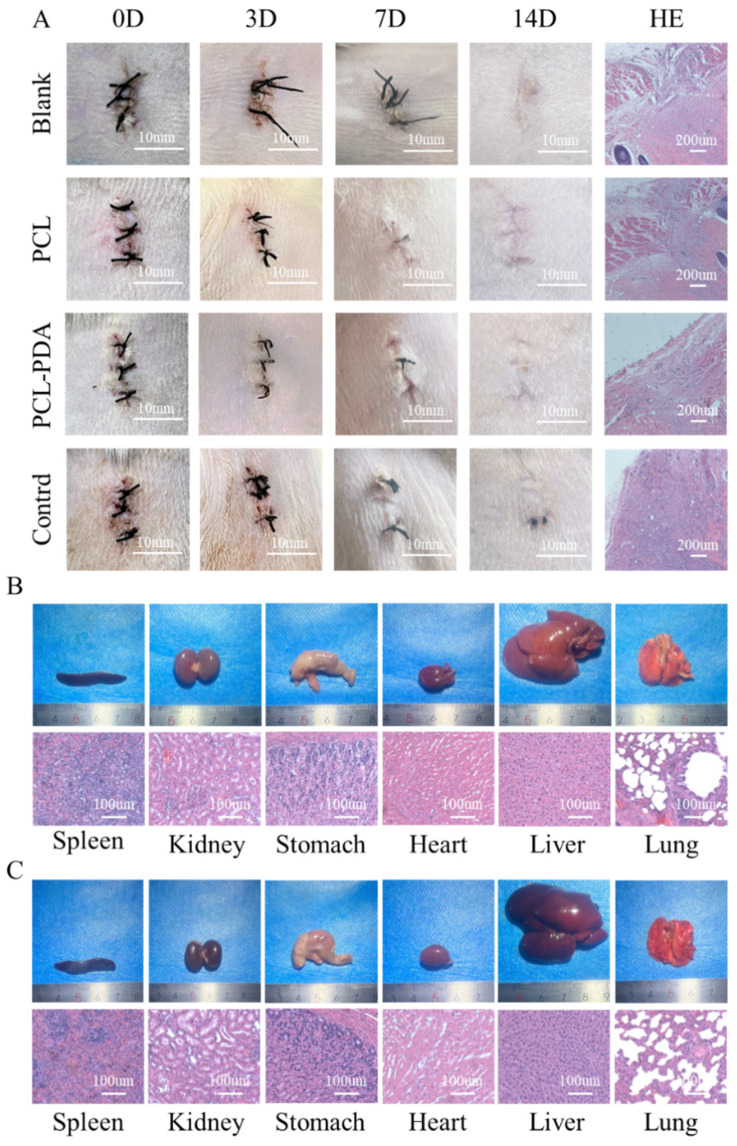
(**A**) Photographs of the wounds that were embedded in the PCL, PCL–PDA, control group and blank group samples immediately following surgery (day 0), and for 3, 7, and 14 days. Histological analysis of subcutaneous tissue of the wounds after embedding surgery at 14 days. Photographs and histological analysis for rat internal organs (heart, lung, stomach, liver, spleen, and kidney) after 14 days embedding experiment with PCL–PDA (**B**) and blank group (**C**), respectively.

To further evaluate the biosafety of the membranes, filter paper (control group), PCL, and PCL–PDA fiber membranes were implanted into the subcutaneous tissue of the rat back (Figure 4A). The images of the wound were recorded after the operation. On the 7th day, the wounds of the control group showed obvious redness and swelling. After 14 days, all wounds recovered completely. HE results showed that the control group had relatively serious foreign body granuloma and inflammatory reaction, while the cells in other groups maintained normal levels, indicating that PCL and PCL–PDA fiber membranes had good biosafety and degradation performance.

In order to further investigate the biotoxic effects of PCL–PDA fiber membranes and their degradation products on other organs, including heart, lung, stomach, liver, spleen, and kidney, we compared the gross and HE results of various organs in SD rats after 14 days of subcutaneous implantation of PCL–PDA fiber membranes. As shown in Figure 4B,C, the shape and size of each organ in the PCL–PDA group did not change significantly, which was similar to that of normal rats. After histological analysis, no substantial damage was found in the organs of the rats treated with PCL–PDA, indicating that there were no obvious side effects on the circulatory system, respiratory system, digestive system, and metabolic system.

### 3.5. Evaluation of In Vitro Blood Clotting of Membranes

**Figure 5:**
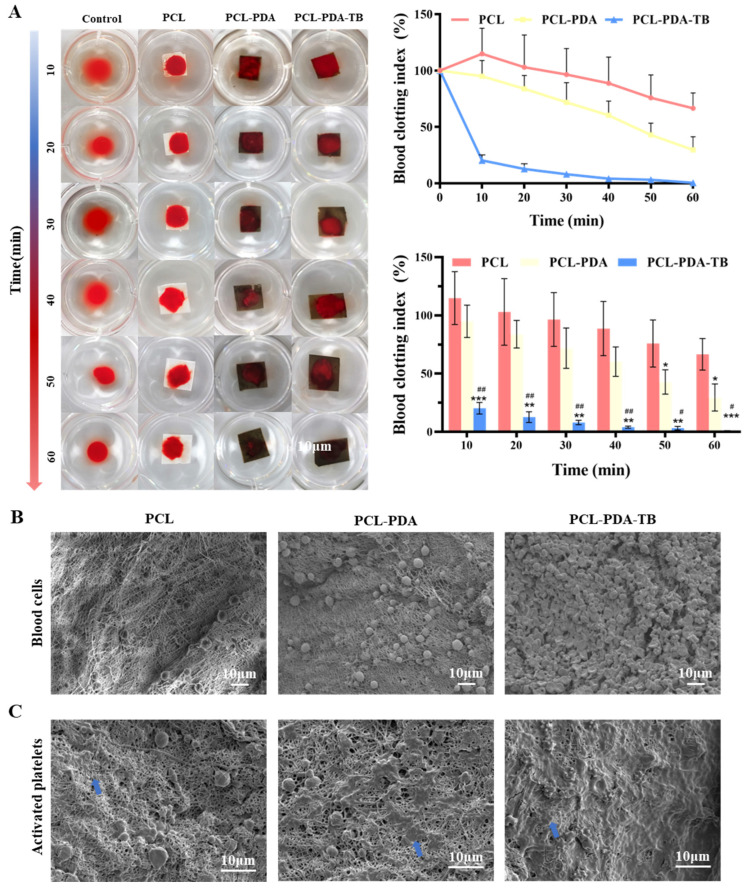
(**A**) Photographs from the in vitro blood-clotting measurement and the blood clotting index of the supernatant absorbance for PCL, PCL–PDA, and PCL–PDA–TB (*n* = 3, * *p* < 0.05, versus PCL group; ** *p* < 0.01, versus PCL group; *** *p* < 0.001, versus PCL group; # *p* < 0.05, versus PCL–PDA group; ## *p* < 0.01, versus PCL–PDA group). The SEM images of blood cells (**B**) and activated platelets (**C**) on the PCL, PCL–PDA, and PCL–PDA–TB surface (blue arrow).

We evaluated the blood coagulability of membranes in vitro. Blood after adding calcium ions was dripped onto the surface of the membrane, and sample photos and BIC were recorded at different time points to measure the coagulation ability of membranes. As shown in Figure 5A, at 10 min, the supernatant clarity of PCL–PDA–TB fiber membrane was significantly higher than that of the control group, PCL, and PCL–PDA groups. This result corresponded to the BCI index result. At 10 min, the BCI of PCL–PDA–TB fiber membrane (20.29 ± 4.05%) was significantly lower than that of PCL fiber membrane (114.83 ± 18.53%, *p* < 0.001) and PCL–PDA fiber membrane (94.96 ± 11.33%, *p* < 0.01). During the whole experiment, the BCI of PCL–PDA–TB membrane was lower than that of PCL and PCL–PDA membrane, indicating that the coagulability and efficiency of PCL–PDA–TB membrane were significantly improved after thrombin loading.

Further, we evaluated the coagulation properties of PCL, PCL–PDA, and PCL–PDA–TB membranes at the microscopic level by SEM images of red blood cell adhesion to platelets. Thrombin can perform hemostasis by converting fibrinogen to fibrin and activating clotting factor XIII to obtain stable fibrin clots. In addition, thrombin activates proteinase-activating receptors on the surface of platelets, causing platelets to activate and promoting platelet adhesion. This is because thrombin on the scaffold causes stable fibrin grids to form quickly in the blood to trap and adsorb more blood material. It can be seen from Figure 5C that compared with PCL and PCL–PDA fiber membranes, there was more activated platelet adhesion on the surface of PCL–PDA–TB. These results indicated that PCL–PDA fiber membranes can maintain thrombin activity and exert coagulation ability after thrombin loading.

### 3.6. Evaluation of Hemostatic Performance In Vivo

**Figure 6:**
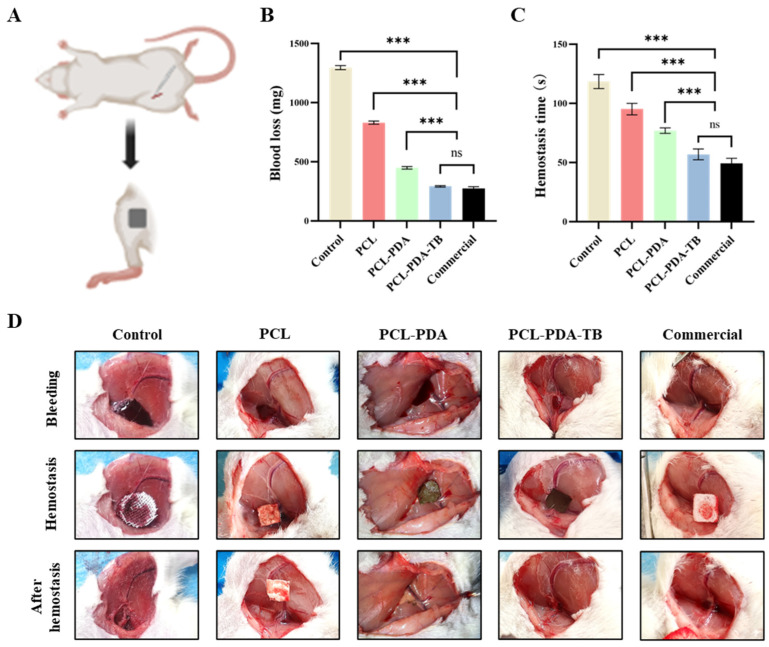
(**A**) Schematic diagram of the rat femoral artery hemostasis model. (**B**) Blood loss and (**C**) hemostatic time on the rat femoral artery hemostasis model by using control, PCL, PCL–PDA, PCL–PDA–TB and commercial. (**D**) Images of the hemostasis (from left to right) by use of control, PCL, PCL–PDA, PCL–PDA–TB, and commercial (*n* = 6, *** *p* < 0.001).

We first tested the hemostatic performance of PCL, PCL–PDA, and PCL–PDA–TB membranes in the femoral artery bleeding model of SD rats (Figure 6A). As can be seen from Figure 6B,C, the amount of blood loss (198.33 ± 2.91 mg) and bleeding time (45.00 ± 1.69 s) in the PCL–PDA–TB group were significantly lower than those in the control group (1099.67 ± 3.14 mg, 106.83 ± 6.43 s, *p* < 0.001), PCL group (775.50 ± 5.41 mg, 66.50 ± 1.50 s, *p* < 0.001), and PCL–PDA group (444.00 ± 13.47 mg, 50.33 ± 1.50 s, *p* < 0.001). There was no significant difference in blood loss and bleeding time between the PCL–PDA–TB group and the commercial group (186.00 ± 4.55 mg, 39.50 ± 0.96 s, *p* > 0.05).

**Figure 7:**
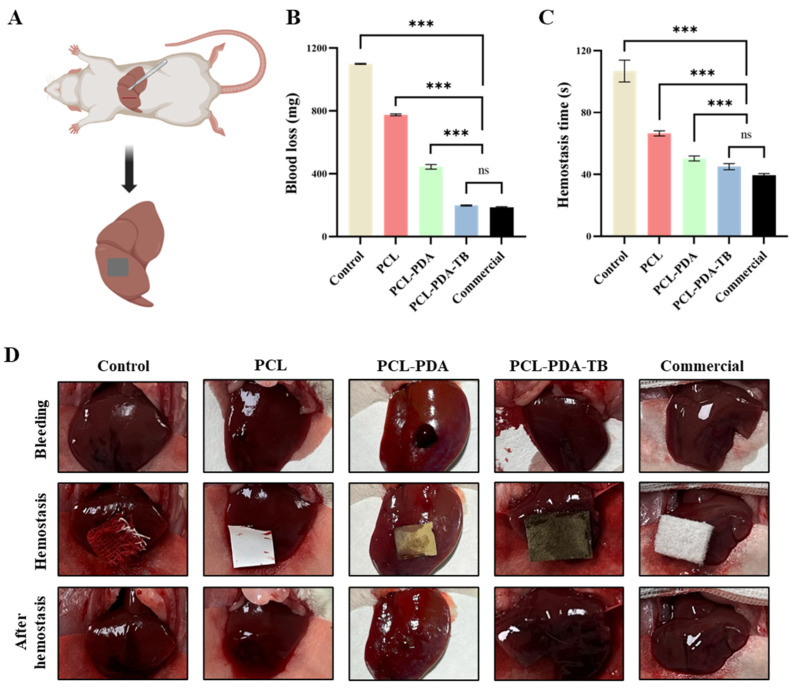
(**A**) Schematic diagram of the rat liver homeostasis model. (**B**) Blood loss and (**C**) hemostatic time on the rat liver homeostasis model by using control, PCL, PCL–PDA, PCL–PDA–TB and commercial. (**D**) Images of the hemostasis (from left to right) by use of control, PCL, PCL–PDA, PCL–PDA–TB and commercial (*n* = 6, *** *p* < 0.001).

Then, we tested the hemostatic performance of PCL, PCL–PDA, and PCL–PDA–TB membranes in a liver dissection model of SD rats (Figure 7A). We found that the amount of blood loss (292.35 ± 5.75 mg) and bleeding time (56.83 ± 4.49 s) in the PCL–PDA–TB group were significantly lower than those in the control group (1296.51 ± 15.28 mg, 118.50 ± 5.44 s). *p* < 0.001), PCL group (831.77 ± 11.67 mg, 95.17 ± 4.49 s, *p* < 0.001), and PCL–PDA group (449.33 ± 9.73 mg, 77.00 ± 2.16 s, *p* < 0.001). There was no significant difference in blood loss volume and bleeding time between the PCL–PDA–TB group and the commercial group (275.34 ± 12.75 mg, 49.33 ± 3.86 s, *p* > 0.05) (Figure 7B,C). The above results indicate that PCL–PDA–TB has good hemostatic performance in vivo, which is close to that of commercial dressings.

## 4. Discussion

The ideal hemostatic material should have the following characteristics: rapid, clear hemostatic effect; portable and easy to make and use; safe and non-toxic, good biocompatibility; and multiple bleeding scenarios [38,39]. Due to the complexity and coordination of coagulation mechanisms, no hemostatic material has been able to fully meet the above hemostatic criteria so far. Therefore, this study designed and prepared a nanofiber membrane packed with thrombin for wound hemostasis.

Thrombin is a coagulation system protease excited by sodium ions that plays a key role in the coagulation cascade [40]. After a traumatic event, thrombin rapidly converts from inactive prothrombin to thrombin and immediately participates in coagulation and hemostasis [41]. Therefore, thrombin is widely used in hemostasis due to its excellent biocompatibility and hemostatic performance. However, because it is not viscous and the powder state is easily dispersed by the blood flow and autolysis, its hemostatic stability is reduced [19,20]. Some studies have tried to incorporate thrombin into hemostatic materials, but failed to solve outstanding problems such as low thrombin load and poor storage conditions [42,43]. In this study, the PCL–PDA loading system was prepared by electrospinning technology and PDA modification strategy, which can increase the thrombin load and stability while ensuring the biological activity of thrombin. By transforming the thrombin powder into a new hemostatic dressing with good hemostatic performance and easy preservation, the advantages of the nanostructure of electrospun fiber and the hemostatic activity of thrombin can be utilized while overcoming the disadvantages of the thrombin powder being easily dispersed. During in vitro experiments, the good microstructure, high thrombin load and biocompatibility of the new hemostatic material were effectively verified by SEM, CLMS, cell proliferation experiment, cell adhesion experiment, and extract cytotoxicity experiment. Erythrocyte adhesion, platelet adhesion, dynamic coagulation time, and animal model experiments proved the good coagulation effect of thrombin nanofiber membrane.

Electrospinning is a common method for preparing hemostatic materials [44,45]. Electrospinning technology mainly includes four parts: high voltage, propel pump, syringe, and receiving device [46]. Nanofibers prepared by electrospinning technology have high porosity, gas permeability, and can provide a high specific surface area [47], which are conducive to removing exudate and achieving hemostasis. Commonly used polymers for hemostatic materials include PEO, PLA, PCL, PVA, etc., often have high mechanical properties, but worse biocompatibility than natural materials [48,49]. In fact, in the actual hemostatic scene, it is difficult to achieve efficient hemostasis by relying only on the physical properties and morphological characteristics of the nanofibers, so adding additional drugs with excellent hemostatic performance is a potential solution.

PDA can simulate the mucous components secreted by marine mussel byssus from the perspective of bionics, and bind to the surface of inorganic, organic, metal, polymer, and other substrates [50]. In addition, the PDA on the surface of the material has many functional groups, such as amino, carboxyl, catechol, and other functional groups, which are easy to form covalent or non-covalent coupling with the amine and mercaptan parts of the protein to achieve efficient loading while maintaining protein activity. Cell surface or protein-like bioactive substances usually have these groups [51]. Studies performed by Ku et al. have demonstrated that PDA did not hinder the viability or proliferation of many kinds of mammalian cells such as fibroblasts, osteoblasts, neurons, and endothelial cells [52]. A lot of investigations have illustrated that PDA-coating even promoted cell adhesion and proliferation on substrates in a material-independent manner compared with the pristine substrates, which further provided strong evidence for the negligible cytotoxicity of PDA [53,54]. In addition, the polydopamine coating is surprisingly stable. Park et al. found that using polydimethylsiloxane as substrate, PDA film can maintain very stable adhesion ability and maintain long-term cell adhesion even under harsh conditions (such as organic solvents, strong acids, ultrasound, and heat treatment) [55]. In this study, dopamine was self-polymerized to form PDA by covalent bond, hydrogen bond, and π–π bond in alkaline environment. PDA has a high content of amines, catechol (3, 4-dichol). The coexistence of these two functional groups contributes to the high adhesion of PDA. This strong adhesion enables the PDA to form a stable PDA coating on the surface of the PCL nanofiber film. As a protein, thrombin has rich amine and mercaptan groups, which can be combined to the surface of PDA by Michael addition or Schiff base reaction, so as to improve the quantity and stability of thrombin load. Through SEM image and surface chemical element analysis, it was found that PDA was successfully coated on the PCL fiber membrane surface without damaging the microstructure of the membrane. The results of water contact showed that the surface hydrophilicity of PCL fiber membrane increased significantly after PDA coating. Studies have shown that hydrophilic surfaces are more conducive to cell adhesion and proliferation [56]. In addition, CLMS observation and SEM image results showed that the thrombin load on PCL fiber membrane increased significantly after PDA coating. These results indicate that the thrombin loading system based on the PDA surface modification has been successfully developed.

Thrombin can convert fibrinogen to fibrin and activate platelets, causing platelet aggregation to promote clotting or hemostasis [57]. In the test of blood coagulation in vitro, we found that PCL–PDA fiber membrane loaded with thrombin can rapidly promote blood coagulation. These results indicate that thrombin can exert normal coagulation function in PCL–PDA loading system, promote the formation of fibrin mesh and platelet adhesion, and form stable thrombus. On the basis of the positive results in vitro, the effect of these membranes on hemostasis in vivo was further evaluated. The PADD-modified PCL fiber membrane is used to connect thrombin to form thrombin membrane, which overcomes the shortcomings of thrombin powder, such as poor strength, easy to fall off, and unstable hemostatic effect caused by premature autolysis [58]. In the experimental model of femoral artery bleeding and liver bleeding in SD rats, PCL–PDA–TB fiber membrane can quickly infiltrate and absorb blood, and maintain a stable shape during hemostasis, so that thrombin can continue to play the hemostatic function. The above properties make the hemostatic performance of PCL–PDA–TB significantly better than the other two membranes, and there is no significant difference with commercial hemostatic dressing. The loading capacity of thrombin on nanofiber membrane was improved by surface modification of polydopamine. It is possible that in the early stage of bleeding, our material increased the release of thrombin at the bleeding site, promoted the conversion of fibrinogen to fibrin, and enhanced platelet activity, thus speeding up the thrombin-related exogenous coagulation reaction and achieving effective hemostasis.

However, this study also has some shortcomings that need to be further improved in the future, mainly including: (1) limited by research conditions as we have not been able to explore the related repair mechanism of hemostasis, which caused the lack of theoretical support, and hemostatic repair mechanism can be an important research direction in the future; (2) the traumatic bleeding models in this study were SD rats, we did not use larger animals such as Bama pigs for further hemostasis verification studies. The application of more advanced traumatic bleeding models and human trials are important research directions for research on hemostatic materials;(3) the small number of animal samples in this study may cause certain bias in the study, and multi-angle multiple hemostatic effect verification will be carried out to further confirm the hemostatic effect of the hemostatic material.

## 5. Conclusions

Hemorrhagic shock is the primary cause of death in all kinds of severe trauma patients. It is of great significance to develop rapid and efficient hemostatic methods to promote the life treatment of trauma patients. In this study, polycaprolactone (PCL) nanofiber membrane was prepared by electrospinning. Inspired by mussel adhesion protein, a PCL–PDA loading system was developed by modifying the surface of polydopamine (PDA), which realized efficient and stable loading of thrombin (TB). The cell experiment and subcutaneous embedding test confirmed that the novel PCL–PDA–TB membrane has good biosecurity. Red cell adhesion, platelet adhesion, dynamic coagulation time, and animal models confirmed that PCL–PDA–TB membrane has a good hemostatic effect. The PCL–PDA–TB membrane prepared in this study can maintain the stable load and biological activity of thrombin, exert the functions of thrombin, and PDA in vitro coagulation experiment, and significantly promote red blood cell adhesion and platelet activation. In conclusion, the PCL–PDA–TB membrane prepared in this study has the advantages of simple preparation method, high hemostatic performance, and low preparation cost, and is expected to be widely used in various traumatic hemostatic scenarios.

## Data Availability

All data that support the findings of this study are included within the article (and any supplementary files).

## Supplementary Materials

The following supporting information can be downloaded at: https://www.mdpi.com/article/10.3390/polym15143122/s1, Figure S1: Elastic modulus of PCL, PCL-PDA and PCL-PDA-TB fibrous membranes.(*n* = 3); Figure S2: Degradation rate of PCL, PCL-PDA and PCL-PDA-TB fibrous membranes.(*n* = 3); Figure S3: SEM images of Thrombin loaded onto PCL and PCL-PDA fibrous membranes; Figure S4: SEM and EDS elemental mapping images of PCL-PDA-TB fibrous membranes.

## References

[1] Champion H.R., Bellamy R.F., Roberts C.P., Leppaniemi A. (2003). A Profile of Combat Injury. J. Trauma Inj. Infect. Crit. Care.

[2] Stewart R.M., Myers J.G., Dent D.L., Ermis P., Gray G.A., Villarreal R., Blow O., Woods B., McFarland M., Garavaglia J. (2003). Seven Hundred Fifty-Three Consecutive Deaths in a Level I Trauma Center: The Argument for Injury Prevention. J. Trauma Inj. Infect. Crit. Care.

[3] Kauvar D.S., Lefering R., Wade C.E. (2006). Impact of Hemorrhage on Trauma Outcome: An Overview of Epidemiology, Clinical Presentations, and Therapeutic Considerations. J. Trauma Inj. Infect. Crit. Care.

[4] Muldowney M., Liu Z., Stansbury L.G., Vavilala M.S., Hess J.R. (2023). Ultramassive Transfusion for Trauma in the Age of Hemostatic Resuscitation: A Retrospective Single-Center Cohort from a Large US Level-1 Trauma Center, 2011–2021. Obstet. Anesth. Dig..

[5] Sperry J.L., Cotton B.A., Luther J.F., Cannon J.W., Schreiber M.A., Moore E.E., Namias N., Minei J.P., Wisniewski S.R., Guyette F.X. (2023). Whole Blood Resuscitation and Association with Survival in Injured Patients with an Elevated Probability of Mortality. J. Am. Coll. Surg..

[6] Wang Y., Wang C., Hu P., Wang H., Gan L., Kong G., Shi Y., Wang T., Jiang B. (2023). China trauma treatment statistics 2019: A national retrospective study based on hospitalized cases. Front. Public Health.

[7] Wang T., Wang Y., Xu T., Li L., Huo M., Li X., He Y., Lin Q., Mei B., Zhou X. (2020). Epidemiological and clinical characteristics of 3327 cases of traffic trauma deaths in Beijing from 2008 to 2017: A retrospective analysis. Medicine.

[8] Gu H., Li H., Wei L., Lu J., Wei Q. (2023). Collagen-based injectable and self-healing hydrogel with multifunction for regenerative repairment of infected wounds. Regen. Biomater..

[9] Ibne Mahbub M.S., Bae S.H., Gwon J.G., Lee B.T. (2023). Decellularized liver extracellular matrix and thrombin loaded biodegradable TOCN/Chitosan nanocomposite for hemostasis and wound healing in rat liver hemorrhage model. Int. J. Biol. Macromol..

[10] Das P., Manna S., Roy S., Nandi S.K., Basak P. (2023). Polymeric biomaterials-based tissue engineering for wound healing: A systemic review. Burn. Trauma.

[11] Lu X., Li X., Yu J., Ding B. (2022). Nanofibrous hemostatic materials: Structural design, fabrication methods, and hemostatic mechanisms. Acta Biomater..

[12] Mecwan M., Li J., Falcone N., Ermis M., Torres E., Morales R., Hassani A., Haghniaz R., Mandal K., Sharma S. (2022). Recent advances in biopolymer-based hemostatic materials. Regen. Biomater..

[13] Mogoşanu G.D., Grumezescu A.M. (2014). Natural and synthetic polymers for wounds and burns dressing. Int. J. Pharm..

[14] Xiao X., Wu Z. (2022). A Narrative Review of Different Hemostatic Materials in Emergency Treatment of Trauma. Emerg. Med. Int..

[15] Zou C.-Y., Li Q.-J., Hu J.-J., Song Y.-T., Zhang Q.-Y., Nie R., Li-Ling J., Xie H.-Q. (2022). Design of biopolymer-based hemostatic material: Starting from molecular structures and forms. Mater. Today Bio.

[16] Valke L.L.F.G., Rijpma S., Meijer D., Schols S.E.M., van Heerde W.L. (2022). Thrombin generation assays to personalize treatment in bleeding and thrombotic diseases. Front. Cardiovasc. Med..

[17] Al-Amer O.M. (2022). The role of thrombin in haemostasis. Blood Coagul. Fibrinolysis.

[18] Jiang S.X., Chahal D., Ali-Mohamad N., Kastrup C., Donnellan F. (2022). Hemostatic powders for gastrointestinal bleeding: A review of old, new, and emerging agents in a rapidly advancing field. Endosc. Int. Open.

[19] Sidonio R.F., Hoffman M., Kenet G., Dargaud Y. (2023). Thrombin generation and implications for hemophilia therapies: A narrative review. Res. Pract. Thromb. Haemost..

[20] Shaw J.R., Castellucci L.A., Siegal D., Carrier M. (2023). DOAC-associated bleeding, hemostatic strategies, and thrombin generation assays—A review of the literature. J. Thromb. Haemost..

[21] Zhang J., Hou Y., Lei L., Hu S. (2022). Moist-electric generators based on electrospun cellulose acetate nanofiber membranes with tree-like structure. J. Membr. Sci..

[22] Yang C., Topuz F., Park S.H., Szekely G. (2022). Biobased thin-film composite membranes comprising priamine–genipin selective layer on nanofibrous biodegradable polylactic acid support for oil and solvent-resistant nanofiltration. Green Chem..

[23] Ji Y., Song W., Xu L., Yu D.-G., Bligh S.W.A. (2022). A Review on Electrospun Poly(amino acid) Nanofibers and Their Applications of Hemostasis and Wound Healing. Biomolecules.

[24] Chen K., Pan H., Ji D., Li Y., Duan H., Pan W. (2021). Curcumin-loaded sandwich-like nanofibrous membrane prepared by electrospinning technology as wound dressing for accelerate wound healing. Mater. Sci. Eng. C.

[25] Liu T., Zhang Z., Liu J., Dong P., Tian F., Li F., Meng X. (2022). Electrospun kaolin-loaded chitosan/PEO nanofibers for rapid hemostasis and accelerated wound healing. Int. J. Biol. Macromol..

[26] Mirmajidi T., Chogan F., Rezayan A.H., Sharifi A.M. (2021). In vitro and in vivo evaluation of a nanofiber wound dressing loaded with melatonin. Int. J. Pharm..

[27] Siddiqui N., Asawa S., Birru B., Baadhe R., Rao S. (2018). PCL-Based Composite Scaffold Matrices for Tissue Engineering Applications. Mol. Biotechnol..

[28] Song L., Ahmed M.F., Li Y., Bejoy J., Zeng C. (2017). PCL-PDMS-PCL Copolymer-Based Microspheres Mediate Cardiovascular Differentiation from Embryonic Stem Cells. Tissue Eng. Part C Methods.

[29] Grossen P., Witzigmann D., Sieber S., Huwyler J. (2017). PEG-PCL-based nanomedicines: A biodegradable drug delivery system and its application. J. Control. Release.

[30] Waite J.H., Tanzer M.L. (1981). Polyphenolic Substance of Mytilus edulis: Novel Adhesive Containing L-Dopa and Hydroxyproline. Science.

[31] Fei F., Le Phuong H.A., Blanford C.F., Szekely G. (2019). Tailoring the Performance of Organic Solvent Nanofiltration Membranes with Biophenol Coatings. ACS Appl. Polym. Mater..

[32] Qin Y., Xu L., Zhu Z., Wong W.Y. (2023). Strongly coupled interface facilitating charge separation to the improved visible light-driven hydrogen production on CdS@ polydopamine/NiS photocatalyst. J. Mater. Chem. A.

[33] Cai S., Zuo X., Zhao H., Yang S., Chen R., Chen L., Zhang R., Ding D., Cai T. (2022). Evaluation of N-doped carbon for the peroxymonosulfate activation and removal of organic contaminants from livestock wastewater and groundwater. J. Mater. Chem. A.

[34] Liu Y., Ai K., Lu L. (2014). Polydopamine and Its Derivative Materials: Synthesis and Promising Applications in Energy, Environmental, and Biomedical Fields. Chem. Rev..

[35] Zhou S., Chang Q., Lu F., Xing M. (2017). Injectable Mussel-Inspired Immobilization of Platelet-Rich Plasma on Microspheres Bridging Adipose Micro-Tissues to Improve Autologous Fat Transplantation by Controlling Release of PDGF and VEGF, Angiogenesis, Stem Cell Migration. Adv. Health Mater..

[36] Lee S.J., Kim M.E., Nah H., Seok J.M., Jeong M.H., Park K., Kwon I.K., Lee J.S., A Park S. (2019). Vascular endothelial growth factor immobilized on mussel-inspired three-dimensional bilayered scaffold for artificial vascular graft application: In vitro and in vivo evaluations. J. Colloid Interface Sci..

[37] Pi W., Zhang Y., Li L., Li C., Zhang M., Zhang W., Cai Q., Zhang P. (2022). Polydopamine-coated polycaprolactone/carbon nanotube fibrous scaffolds loaded with brain-derived neurotrophic factor for peripheral nerve regeneration. Biofabrication.

[38] Muzzarelli R.A. (2009). Chitins and chitosans for the repair of wounded skin, nerve, cartilage and bone. Carbohydr. Polym..

[39] Pendharkar S., Jian G. (2004). Biodegradable Hemostatic Wound Dressings. U.S. Patent.

[40] Mısırlıoğlu S., Türkgeldi E., Yagmur H., Urman B., Ata B. (2018). Use of a gelatin-thrombin hemostatic matrix in obstetrics and gynecological surgery. J. Turk. Soc. Obstet. Gynecol..

[41] Wasilko S.M., Quinlan N.J., Shafritz A.B. (2015). Topical Hemostatic Agents and Their Role in Upper Extremity Surgery. J. Hand Surg..

[42] Lawson J.H. (2006). The Clinical Use and Immunologic Impact of Thrombin in Surgery. Semin. Thromb. Hemost..

[43] Takeda K. (2004). Role of increase in permeability and circulatory failure in the development of organ dysfunction in severe acute pancreatitis. Nihon Rinsho. Jpn. J. Clin. Med..

[44] Sánchez-Machado D.I., Maldonado-Cabrera A., López-Cervantes J., Maldonado-Cabrera B., Chávez-Almanza A.F. (2023). Therapeutic effects of electrospun chitosan nanofibers on animal skin wounds: A systematic review and meta-analysis. Int. J. Pharm. X.

[45] Pilehvar-Soltanahmadi Y., Dadashpour M., Mohajeri A., Fattahi A., Sheervalilou R., Zarghami N. (2018). An Overview on Application of Natural Substances Incorporated with Electrospun Nanofibrous Scaffolds to Development of Innovative Wound Dressings. Mini-Rev. Med. Chem..

[46] Xue J., Wu T., Dai Y., Xia Y. (2019). Electrospinning and Electrospun Nanofibers: Methods, Materials, and Applications. Chem. Rev..

[47] Elangwe C.N., Morozkina S.N., Olekhnovich R.O., Polyakova V.O., Krasichkov A., Yablonskiy P.K., Uspenskaya M.V. (2023). Pullulan-Based Hydrogels in Wound Healing and Skin Tissue Engineering Applications: A Review. Int. J. Mol. Sci..

[48] Petroni S., Tagliaro I., Antonini C., D’arienzo M., Orsini S.F., Mano J.F., Brancato V., Borges J., Cipolla L. (2023). Chitosan-Based Biomaterials: Insights into Chemistry, Properties, Devices, and Their Biomedical Applications. Mar. Drugs.

[49] Erdi M., Sandler A., Kofinas P. (2023). Polymer nanomaterials for use as adjuvant surgical tools. WIREs Nanomed. Nanobiotechnol..

[50] Zhao H., Waite J.H. (2006). Linking Adhesive and Structural Proteins in the Attachment Plaque of Mytilus californianus. J. Biol. Chem..

[51] Yang S.H., Kang S.M., Lee K.-B., Chung T.D., Lee H., Choi I.S. (2011). Mussel-Inspired Encapsulation and Functionalization of Individual Yeast Cells. J. Am. Chem. Soc..

[52] Ku S.H., Ryu J., Hong S.K., Lee H., Park C.B. (2010). General functionalization route for cell adhesion on non-wetting surfaces. Biomaterials.

[53] Ku S.H., Lee M., Park C.B. (2013). Carbon-Based Nanomaterials for Tissue Engineering. Adv. Healthc. Mater..

[54] Luo R., Tang L., Zhong S., Yang Z., Wang J., Weng Y., Tu Q., Jiang C., Huang N. (2013). In Vitro Investigation of Enhanced Hemocompatibility and Endothelial Cell Proliferation Associated with Quinone-Rich Polydopamine Coating. ACS Appl. Mater. Interfaces.

[55] Ku S.H., Lee J.S., Park C.B. (2010). Spatial Control of Cell Adhesion and Patterning through Mussel-Inspired Surface Modification by Polydopamine. Langmuir.

[56] Wu R., Gao G., Xu Y. (2020). Electrospun Fibers Immobilized with BMP-2 Mediated by Polydopamine Combined with Autogenous Tendon to Repair Developmental Dysplasia of the Hip in a Porcine Model. Int. J. Nanomed..

[57] Dimitroulis D., Antoniou E., Karidis N.P., Kontzoglou K., Kouraklis G. (2012). Surgical control of life-threatening post-ERCP bleeding with a gelatin matrix-thrombin hemostatic agent. Int. J. Surg. Case Rep..

[58] Coughlin S.R. (1999). Protease-activated receptors and platelet function. Thromb. Haemost..

