# Supplementary material for: Polydopamine-Coated Polycaprolactone Electrospun Nanofiber Membrane Loaded with Thrombin for Wound Hemostasis
Source: Polymers (Basel). 2023 Jul 22;15(14):3122. doi: 10.3390/polym15143122 (PMC10385294; doi:10.3390/polym15143122)
Supplement: Supplementary file 1 [file polymers-15-03122-s001.zip › polymers-2477535-supplementary.pdf]

Supplementary Materials

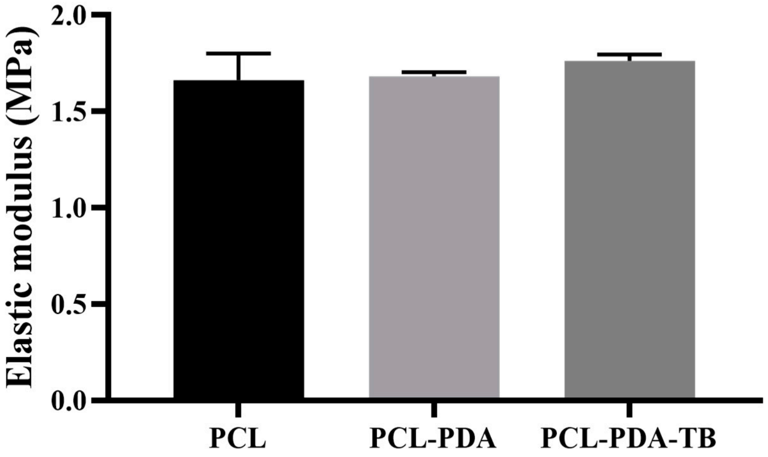

Figure S1. Elastic modulus of PCL, PCL-PDA and PCL-PDA-TB fibrous membranes.(n=3)

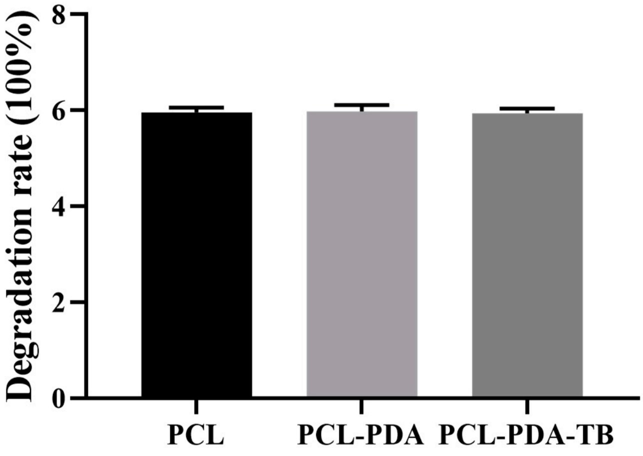

Figure S2. Degradation rate of PCL, PCL-PDA and PCL-PDA-TB fibrous membranes.(n=3)

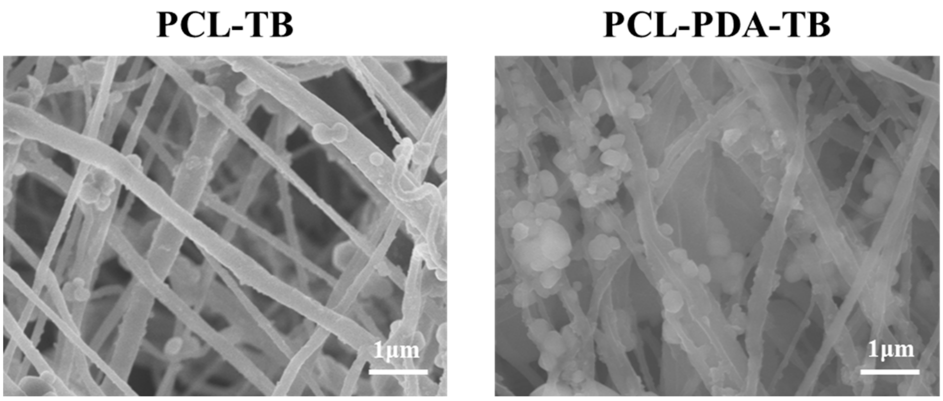

Figure S3. SEM images of Thrombin loaded onto PCL and PCL-PDA fibrous membranes.

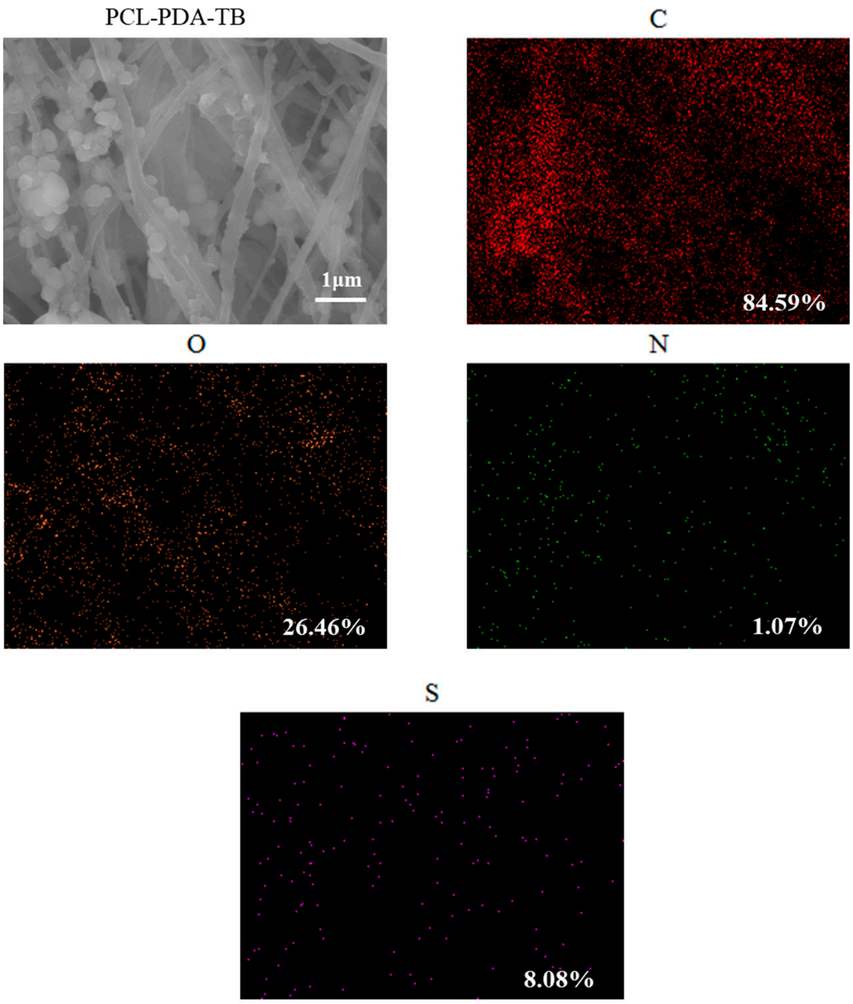

Figure S4. SEM and EDS elemental mapping images of PCL-PDA-TB fibrous membranes.
